# Real-world retrospective study of repetitive transcranial magnetic stimulation (TMS) treatment for bipolar and unipolar depression using TMS registry data in Tokyo

**DOI:** 10.1016/j.heliyon.2024.e27288

**Published:** 2024-03-04

**Authors:** Haruki Ikawa, Ryota Osawa, Yuya Takeda, Akiko Sato, Hoshimi Mizuno, Yoshihiro Noda

**Affiliations:** a Tokyo Yokohama TMS Clinic, Tokyo, Japan; b Department of Neuropsychiatry, Keio University School of Medicine, Tokyo, Japan

**Keywords:** Bipolar depression (BD), Dorsolateral prefrontal cortex (DLPFC), Intermittent theta burst stimulation (iTBS), Low-frequency repetitive transcranial magnetic stimulation (LF-rTMS), Unipolar depression (MDD)

## Abstract

Despite the prevalence of empirical practice, evidence supporting the use of repetitive transcranial magnetic stimulation (rTMS) in treating bipolar depression (BD) is sparse compared to that for unipolar depression. Therefore, this study aimed to conduct a retrospective observational analysis using TMS registry data to compare the efficacy of rTMS treatment for BD and unipolar depression. Data from 20 patients diagnosed with unipolar and BD were retrospectively extracted from the TMS registry to ensure age and sex matching. The primary outcomes of this registry study were measured using the 21-item Hamilton Depression Rating Scale (HAM-D_21_) and Montgomery−Åsberg Depression Rating Scale (MADRS). Analysis did not reveal significant differences between the two groups in terms of depression severity, motor threshold, or stimulus intensity at baseline. Similarly, no significant differences were observed in absolute or relative changes in the total HAM-D_21_ and MADRS scores. Furthermore, the response and remission rates following rTMS treatment did not differ significantly between groups. The only adverse event reported in this study was scalp pain at the stimulation site; however, the incidence and severity were not significantly different between the groups. In conclusion, this retrospective study, using real-world TMS registry data, suggests that rTMS treatment for BD could be as effective as that for unipolar depression. These findings underscore the need for further validation in prospective randomized controlled trials with larger sample sizes.

## 1 Introduction

Bipolar disorder (BD), which affects approximately 2% of the population [1], is a debilitating condition that significantly impairs quality of life and functional capacity. It is associated with a higher incidence of comorbidities and mortality than in healthy individuals [2]. This distressing illness imposes a psychosocioeconomic burden due to recurrent depressive episodes [3,4]. Furthermore, BD is often characterized by refractory cases that do not respond adequately to pharmacotherapy [5]. Given this context, the development of innovative therapeutic strategies for BD is highly anticipated.

Repetitive transcranial magnetic stimulation (rTMS) is a therapeutic modality that stimulates specific cortical areas and exerts therapeutic effects through neuromodulation of the stimulated site and its interconnected networks [6]. In particular, high-frequency (HF) rTMS and intermittent theta burst stimulation (iTBS) applied to the left dorsolateral prefrontal cortex (DLPFC) can improve depressive symptoms among patients with treatment‐resistant unipolar depression [7,8]. Meanwhile, low-frequency (LF) rTMS to the right DLPFC has been shown to yield antidepressant effects comparable to those of HF-rTMS to the left DLPFC in a mixed cohort of patients with unipolar depression and BD [9,10]. This protocol also mitigates pain at the stimulation site in healthy subjects [11] and reduces the risk of seizure induction in patients with refractory partial epilepsy [12]. In this context, HF-left DLPFC and LF-right DLPFC rTMS have emerged as promising therapeutic strategies for BD.

Goldwaser et al. analyzed the database of their own rTMS service to identify patients diagnosed with bipolar I or II disorder who were in the depressive phase at the time of initiation of rTMS. They examined response and remission rates based on MADRS scores, time to onset of the effect, and any adverse events of manic conversion induced by rTMS treatment [13]. In their study, all patients with BD had previously not responded to at least two antidepressants and were on at least one mood stabilizer at the time of entry into the study, with no concurrent use of antidepressants. rTMS was administered to the left DLPFC at a stimulus intensity of 120% of the motor threshold, with the other parameters set at 10 Hz × 4 s, a 26 s intertrain interval, and 75 trains (37.5 min/session), totaling 3000 pulses in five sessions per week for 30 treatments (or until remission criteria were met). Consequently, for BD as a whole, 77% of the patients met the response criteria and 41% met the remission criteria. These results were numerically superior to the 62% response and 31% remission rates observed for unipolar depression. In general, the treatment was well-tolerated and showed good efficacy. Treatment-induced emotional conversion has not been documented according to clinical criteria [13].

McGirr et al. conducted a literature review of randomized, double-blind sham-controlled trials (RCT) on rTMS for mood disorders. In these studies, patients with BD were randomly assigned to the active or sham rTMS group and underwent a minimum of five rTMS intervention sessions [14]. Their findings indicated a trend toward differential efficacy depending on the stimulation target. Specifically, RCTs that targeted the right DLPFC demonstrated remarkable efficacy, with 60.0% of active rTMS patients achieving a clinical response compared to 6.6% of sham rTMS patients. Furthermore, the incidence of manic conversion attributed to rTMS treatment was extremely low, with no increased risk of active rTMS (0.9% vs. 1.3%; p = 0.97). They concluded that rTMS could be a safe and effective treatment for acute BD with a degree of efficacy comparable to that observed in patients with major depressive disorder. The authors further suggested that rTMS protocols targeting the right DLPFC with an inhibitory LF or continuous TBS (cTBS) may be particularly effective [14]. Nguyen et al. conducted a systematic review of sham-controlled RCT trials investigating rTMS for BD. However, when they separately analyzed the stimulation protocols, statistically significant clinical responses were only observed with HF-rTMS to the left DLPFC (OR: 2.57; 95% CI: 1.17−5.66) [15].

Subsequently, McGirr et al. conducted a two-center, RCT, administering daily active or sham iTBS to the left DLPFC at a stimulus intensity of 120% of the resting motor threshold for 4 weeks. They found that iTBS was not effective in treating acute BD in patients concurrently receiving antimanic or mood-stabilizing agents [16]. Specifically, in their RCT, the clinical response rate in the active iTBS group for BD was 16.7%, a figure considerably lower than the clinical response rate exceeding 40% [8,17] observed in patients with active iTBS for major depressive disorder (MDD).

To date, only a limited number of RCTs have been conducted to investigate the efficacy of rTMS treatment exclusively in patients with BD, and definitive results remain elusive. In particular, in Japan, there is a lack of studies that have adequately assessed the efficacy of rTMS for patients with BD using objective clinical rating scales.

Therefore, we conducted a retrospective observational study using the real-world TMS registry data obtained from our clinic. In this context, real-world data encompass all data procured from clinical sites, specifically clinical data derived from medical practices obtained in clinical settings, such as receipts and electronic medical records. In this study, we specifically scrutinized registry data under the premise that the outcomes of treatment with LF-rTMS for the right DLPFC in patients with BD would parallel the results of iTBS treatment for the left DLPFC, which is generally administered to patients with unipolar depression and would exhibit a certain degree of efficacy.

## 2 Materials and methods

### 2.1 Patient data attributes

In this retrospective observational study, we analyzed data from 20 outpatients with BD (11 males and nine females, mean age 36.8 ± 11.6 years) and 20 patients with unipolar depression (11 males and nine females, mean age 37.3 ± 11.3 years). These patients were retrospectively matched for age (±3 years allowed) and sex, and their data were extracted from the TMS registry for the analysis (Table 1). The study only included data from patients who had been diagnosed with bipolar or MDD at their referral psychiatric facility and had a DSM-5 diagnosis of bipolar or unipolar depression confirmed by certified psychiatrists during their initial visit to the Tokyo Yokohama TMS Clinic. This TMS registry and retrospective observational analysis study received approval from the ITO Yoyogi Mental Clinic Research Ethics Committee (ID: RKK319) and was conducted in accordance with the Ethical Guidelines for Medical and Health Research Involving Human Subjects and the Declaration of Helsinki at Tokyo Yokohama TMS Clinic. According to the study protocol of the TMS registry, informed consent was obtained for prospective data, while an opt-out procedure was used for past data.

**Table 1:** Clinico-demographics at baseline for patients with bipolar depression and unipolar depression at baseline.

	Bipolar depression	Unipolar depression	Statistics for group comparison
N	20	20	
Male	11	11	
Female	9	9	
Age (mean ± S.D.) [years]	36.8 (±11.6)	37.3 (±11.3)	t_38_ = 0.14, p = 0.89
Age of onset [years]	33.1 (±11.4)	32.3 (±11.9)	t_38_ = 0.20, p = 0.84
Duration of illness [years]	4.4 (±4.9)	6.5 (±7.2)	t_38_ = −1.06, p = 0.30
Maudsley staging method	6.7 (±1.1)	6.2 (±1.0)	t_38_ = 1.33, p = 0.19
Resting motor threshold [%]	56.0 (±7.0)	53.6 (±8.4)	t_38_ = 0.96, p = 0.34
Stimulus intensity [%]	66.7 (±8.6)	64.1 (±9.9)	t_38_ = 0.88, p = 0.38
HAM-D_21_ baseline	21.4 (±3.5)	19.5 (±4.8)	t_38_ = 1.40, p = 0.17
MADRS baseline	31.4 (±6.4)	30.2 (±4.4)	t_38_ = 0.69, p = 0.49

S.D. = standard deviation

### 2.2 Data Extraction criteria for the present study

The eligibility criteria for this study were as follows: (1) patients aged ≥18 years; (2) met the Diagnostic and Statistical Manual of Mental Disorders, 5th edition (DSM-5) diagnostic criteria for MDD or BD, as confirmed by standard psychiatric consultation with certified psychiatrists; (3) exhibited persistent depressive symptoms despite undergoing a period of standard pharmacotherapy; (4) had no prior history of convulsive seizures; and (5) had no other discernible contraindications to TMS treatment.

### 2.3 rTMS treatment protocols used in this study

To treat unipolar depression, the original iTBS protocol was applied to the left DLPFC [8]. Conversely, LF rTMS has been administered to the right DLPFC of patients with BD [18]. Specifically, in the case of unipolar depression, the left DLPFC was targeted and stimulated with a double dose of iTBS (1200 pulses, 6 min) at a stimulus intensity of 120% of the resting motor threshold (RMT) [19,20]. In contrast, for BD, 1 Hz LF rTMS (600 pulses, 10 min) was administered at 120% RMT stimulation intensity, targeting the right DLPFC [21]. The rationale for using LF rTMS for the right DLPFC in patients with BD was the higher risk of manic conversion associated with rTMS treatment in BD compared to unipolar disorder [12] and the potential efficacy of an inhibitory LF-rTMS protocol targeting the right DLPFC for BD, as suggested by the study conducted by McGirr et al. [14]. In the dataset used in the current study, all patients underwent 30 sessions of iTBS or rTMS.

The target site on the left DLPFC of each patient was identified using the Beam F3 method [22]. The target site on the right DLPFC was determined by inverting the coordinates of the left DLPFC from left to right. For the rTMS treatment, we used MagPro R30 TMS devices equipped with a Cool-B65 TMS coil or a Cool-B70 TMS coil (MagVenture, Farum, Denmark).

### 2.4 Clinical assessments

Clinical evaluations in this study were performed in patients with bipolar and unipolar depression both before and after a regimen of 30 rTMS treatment sessions. These evaluations used the Hamilton Depression Rating Scale 21-items (HAM-D_21_) and the Montgomery−Åsberg Depression Rating Scale (MADRS). In this study, only data from patients who consented to participate in the TMS registry study (Noda et al., 2022) were used. The response was defined as a ≥50% reduction in HAM-D_21_ or MADRS scores before and after rTMS treatment. Meanwhile, remission was defined as a score of ≤10 on both HAM-D_21_ and MADRS at the end of the treatment. Table 1 presents the mean scores (±S.D.) of the HAM-D_21_ and MADRS at baseline. In addition, treatment resistance was assessed using the Maudsley staging method [23].

### 2.5 Statistical analysis

Statistical analyses were performed using SPSS software (International Business Machines Corporation, Armonk, NY, USA; version 28.0). First, the Shapiro−Wilk test was used to confirm the normality of the clinical data. Subsequently, depending on the nature of the data, group comparisons were performed using the chi-square test for categorical data and the *t*-test for continuous data. The level of significance was set at p < 0.05.

## 3 Results

There were no discernible differences in baseline clinical and epidemiological characteristics, specifically age, resting motor threshold, and stimulus intensity, between patients diagnosed with BD and those with unipolar depression (Table 1).

Absolute and relative changes in total scores on HAM-D_21_ (Table 2) and MADRS (Table 3) did not reveal significant differences between the two groups in terms of treatment efficacy. Furthermore, there were no notable differences in response and remission rates based on the total scores of HAM-D_21_ and MADRS (Table 2, Table 3).

**Table 2:** Treatment efficacy in patients with bipolar depression and unipolar depression assessed by HAM-D_21_

	Bipolar depression	Unipolar depression	Statistics for group comparison
ΔHAMD_21_	13.2 (±4.6)	12.3 (±3.7)	t_38_ = 0.68, p = 0.50
HAMD_21_ improvement rate (%)	61.8 (±19.9)	63.8 (±16.5)	t_38_ = 0.36, p = 0.73
HAMD_21_ response rate (%) (response/non-response)	75 (15/5)	85 (17/3)	χ^2^ (1) = 0.63, p = 0.43
HAMD_21_ remission rate (%) (remission/non-remission)	70 (14/6)	80 (16/4)	χ^2^ (1) = 0.53, p = 0.47

ΔHAM-D_21_ = HAM-D_21_ baseline – HAM-D_21_ post

**Table 3:** Treatment efficacy in patients with bipolar depression and unipolar depression as assessed by MADRS

	Bipolar depression	Unipolar depression	Statistics for group comparison
ΔMADRS	20.9 (±8.4)	19.7 (±4.6)	t_38_ = 0.56, p = 0.58
%changes in MADRS score (%)	65.6 (±22.5)	68.3 (±16.9)	t_38_ = 0.42, p = 0.68
MADRS response rate (%) (response/non-response)	80 (16/4)	90 (18/2)	χ^2^ (1) = 0.78, p = 0.38
MADRS remission rate (%) (remission/non-remission)	55 (11/9)	60 (12/8)	χ^2^ (1) = 0.10, p = 0.75

ΔMADRS = MADRS baseline - MADRS post

The adverse events noted in this data analysis were limited to mild pain at the stimulation site. Chi-square tests performed to determine the incidence of this adverse event in patients with bipolar and unipolar depression did not indicate significant differences between the two groups (Table 4). Other serious adverse events, such as seizures or hypomanic/manic changes, were not reported.

**Table 4:** Differences in the incidence of adverse events between patients with bipolar and unipolar depression

	Bipolar depression	Unipolar depression	Statistics for group comparison
Stimulation site pain (%) (Number of Yes/No)	20 (4/16)	25 (5/15)	χ^2^ (1) = 0.14, p = 0.71

## 4 Discussion

This analytical study was the first retrospective observational study in Japan to evaluate the effectiveness of rTMS treatment for bipolar and unipolar depression using empirical real-world data derived from the TMS registry. The outcomes of rTMS treatment in a cohort of 20 patients, each with BD and 20 patients with unipolar depression, retrospectively matched for age, sex, and baseline severity of depressive symptoms, indicated a significant amelioration of depressive symptoms in both groups, as assessed using the HAM-D_21_ and MADRS scores, which were the primary outcomes. Furthermore, the degree of improvement exhibited parity in both groups.

The antidepressant effect of rTMS treatment on BD, as observed in this data analysis, was found to be on par with that of unipolar depression, corroborating previous reports [21,24,25]. In contrast, Yang et al. posited that patients with BD are less likely to achieve a clinical response than those with unipolar depression [26]. Previous studies on rTMS treatment for BD have used various rTMS protocols, including HF-rTMS to the left DLPFC, LF-rTMS to the right DLPFC, sequential bilateral stimulation (BL)-rTMS to the BL-DLPFC, iTBS to the left DLPFC, and cTBS to the right DLPFC, which could potentially explain the heterogeneity of treatment outcomes. Furthermore, to date, the majority of rTMS clinical studies for mood disorders have included unipolar and BD in their study populations [27], with only a handful of rTMS clinical studies conducted exclusively in patients with BD, thus complicating the interpretation of the results of these rTMS studies.

Although numerous rTMS treatment protocols have been substantiated for their efficacy in treating unipolar depression, as corroborated by a multitude of previous studies [[28], [29], [30]], few rTMS treatment protocols have been rigorously validated for their effectiveness in treating BD. In a systematic review of RCTs that examined the effectiveness of rTMS treatment for BD conducted by Nguyen et al., the only rTMS protocol exhibiting a significant therapeutic effect was HF-rTMS to the left DLPFC [15], while LF-rTMS to the right DLPFC and rTMS to the bilateral DLPFC were empirically shown to be effective. However, the small sample size of each study limits the ability to draw definitive conclusions. A recent systematic review and meta-analysis of RCTs on BD by Kishi et al. deduced that rTMS for the bilateral DLPFC was effective and deep TMS and iTBS for the left DLPFC exhibited a tendency toward improvement in patients with BD [31]. Therefore, previous research does not appear to provide statistically robust results on the efficacy of LF-rTMS of the right DLPFC for BD, which is potentially attributable to the limited number of studies that validate its efficacy. However, in real-world clinical practice, we often observe that the LF-rTMS of the right DLPFC in patients with BD exerts relatively stable and robust effects.

Given that most previous studies on BD have predominantly evaluated the efficacy of HF-rTMS for the left DLPFC [18], the strength of this observational study, using data from the TMS registry contrasting rTMS treatment outcomes in age- and sex-matched patients with unipolar depression, lies in its examination of the effectiveness of LF-rTMS treatment for the right DLPFC in patients with BD. Furthermore, the benefit of ensuring the efficacy of LF-rTMS for the right DLPFC in BD is that since the rTMS protocol has a lower risk of manic changes than the HF-rTMS protocol for the left DLPFC [18], rTMS treatment can be administered more safely in this population. In the present study, there were no significant differences in the incidence of adverse events between patients with bipolar and unipolar depression.

In particular, a single-blind RCT conducted by Mak et al., using 1 Hz LF-rTMS for the right DLPFC in patients with BD who did not respond to antidepressants, with a regimen of 15 sessions that included 300 pulses per session at a stimulation intensity of 110% RMT, did not yield a significant treatment effect [32]. However, the present study, which used more intense stimulus parameters, including 120% RMT for stimulus intensity, 600 pulses per session, and 30 sessions, may explain the enhanced antidepressant effect observed, in contrast to the study by Mak et al. Future investigations should explore the effectiveness of LF-rTMS in the right DLPFC to determine whether further intensification or optimization of each parameter can yield superior antidepressant effects. Given the scarcity of high-quality RCT that evaluate the effectiveness of LF-rTMS to the right DLPFC in BD [33], there is a pressing need for validation studies employing RCT designs with sufficient sample sizes.

This study has some limitations. First, since the TMS registry project [34] was started 3 years ago, the sample size within the registry database was somewhat restricted. Therefore, it is necessary to expand the sample size and conduct large-scale registry data analyses in the future, including case–control studies under more controlled conditions. Second, the retrospective observational nature of this study excludes the possibility of incorporating a placebo control group using sham stimulation treatment. A prospective RCT of BD, which is currently in progress in Japan, is expected to provide rigorous validation. Third, the response rates in the current study, evaluated using MADRS and HAM-D_21_, were approximately 80% for both BD and unipolar depression, suggesting a potential placebo effect. This could be attributed to the fact that the present study was an observational study with a real-world, open-label design and that the number of medical institutions in Japan offering rTMS treatment was limited at the time of the study. Consequently, the expectations of rTMS treatment may have contributed significantly to the placebo effect.

## 5 Conclusion

In this retrospective observational study that used real-world registry data and ensured a matched baseline clinicodemographic background between patients with bipolar and unipolar depression, rTMS treatment showed comparative benefits in both groups. More comprehensive studies with larger sample sizes are required to confirm the effectiveness of LF-rTMS to the right DLPFC in patients with BD.

## Ethical statement

This study complied with the tenets of the Declaration of Helsinki and was approved by the local Research Ethics Board. In accordance with the study protocol of the TMS registry, informed consent was obtained from prospective participants, and an opt-out procedure was applied to past data.

## Data availability

The data analyzed in the present study are available from the corresponding author upon reasonable request.

## Additional information

No additional information is available for this paper.

## CRediT authorship contribution statement

**Haruki Ikawa:** Writing – original draft, Investigation, Formal analysis, Data curation. **Ryota Osawa:** Writing – original draft, Validation, Resources, Investigation. **Yuya Takeda:** Validation, Project administration, Investigation. **Akiko Sato:** Investigation, Formal analysis, Data curation. **Hoshimi Mizuno:** Project administration, Investigation. **Yoshihiro Noda:** Writing – review & editing, Validation, Supervision, Project administration, Methodology, Investigation, Conceptualization.

## Declaration of competing interest

The authors declare that they have no known competing financial interests or personal relationships that could have appeared to influence the work reported in this paper.
